# Supplementary material for: Ellipsoid Localization Microscopy Infers the Size and Order of Protein Layers in Bacillus Spore Coats
Source: Biophys J. 2015 Nov 17;109(10):2058–66. doi: 10.1016/j.bpj.2015.09.023 (PMC4656864; doi:10.1016/j.bpj.2015.09.023)
Supplement: Document S1. Supporting Materials and Methods and one table [file mmc1.pdf]

Biophysical Journal

Supporting Material

Ellipsoid Localization Microscopy Infers the Size and Order of Protein Layers in *Bacillus* Spore Coats

Julia Manetsberger,[1] James D. Manton,[1] Miklos J. Erdelyi,[2] Henry Lin,[1] David Rees, Graham Christie,[1] and Eric J. Rees[1,]*

[1]Department of Chemical Engineering and Biotechnology, University of Cambridge, Cambridge, United Kingdom; and [2]Department of Optics and Quantum Electronics, University of Szeged, Szeged, Hungary

Supporting Information 1

Table S1 Bacterial strains used in this study

**Strain**	**Relevant genotype** [a]	**Source/reference**
B. megaterium strains		
QM B1551	Wild-type strain	P.S. Vary
GFP fusion strains		
JM100	BMQ_0737::pVLG6 (*gfp*) Cm^r	1
JM101	BMQ_3035::pVLG6 (*gfp*) Cm^r	1
JM102	BMQ_4051::pVLG6 (*gfp*) Cm^r	1
JM103	*cotE*::pVLG6 (*gfp*) Cm^r	1
JM104	pHT-*cotX1-gfp* MLS^r	This study
JM105	pHT-*cotX2-gfp* MLS^r	This study
FIU104	*sleL*::pVLG6 (*gfp*) Cm^r	2
B. subtilis strains		
168 1A1	Wild-type strain *TrpC2*	*Bacillus* Genetic Stock Centre
HL100	*TrpC2 amyE::sleL-gfp* Cm^r	This study
HL101	*TrpC2 amyE::cotG-gfp* Cm^r	This study
HL102	*TrpC2 amyE::cotZ-gfp* Cm^r	This study

[a] Abbreviations for antibiotics: Cm^r, chloramphenicol resistance (5 µg/ml); MLSr, lincomycin (25 µg/ml) and erythromycin (1 µg/ml).

References

1. Manetsberger, J., Hall, E. A., and Christie, G. (2014) BMQ_0737 encodes a novel protein crucial to the integrity of the outermost layers of *Bacillus megaterium* QM B1551 spores. *FEMS microbiology letters* **358**, 162-169.

2. Üstok, F. I., Packman, L. C., Lowe, C. R., and Christie, G. (2014) Spore germination mediated by *Bacillus megaterium* QM B1551 SleL and YpeB. *Journal of bacteriology* **196**, 1045-1054.

Supporting Information 2

Image analysis software for the paper:
Ellipsoid localisation microscopy infers the size and order of protein layers in *Bacillus* spore coats

Software Authors
J.D. Manton[1], E.J. Rees[1]*
1 Department of Chemical Engineering and Biotechnology, University of Cambridge, CB2 3RA, UK.
* Correspondence ejr36@cam.ac.uk

This software is open source, and can be obtained from the following website (or alternatively by contacting Eric Rees with the correspondence address above). This source may be updated from time to time.
http://laser.ceb.cam.ac.uk/research/our-software

A version of the software from the time of publication is archived at data.cam.ac.uk/

LINK: https://www.repository.cam.ac.uk/handle/1810/251135

reference ID: [opendata #2026] at info@data.cam.ac.uk

Supporting Information 3: Model leading to equation 4 (spherical shell image)

Algebraic model for image of a spherical spore shell

EJR/HDR.

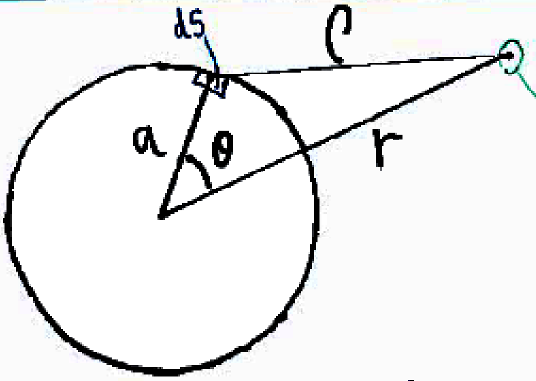

$f(r)$ = Image brightness at this point
ρ = Distance to area element dS
a = Spherical shell radius.

(1) $$\rho^2 = r^2 + a^2 - 2ar\cos\theta. \quad \text{(Cosine rule)}$$

(2) $$\underbrace{df}_{\substack{\text{CONTRIBUTION TO} \\ \text{IMAGE INTENSITY} \\ f(r)}} = \underbrace{(I_0\, dS)}_{\substack{\text{FLUORESCENCE} \\ \text{EMITTED} \\ \text{FROM AREA} \\ \text{ELEMENT } dS}} \times \underbrace{\frac{e^{-\rho^2/2\sigma^2}}{(2\pi\sigma^2)}}_{\substack{\text{SPHERICALLY SYMMETRIC} \\ \text{GAUSSIAN POINT SPREAD} \\ \text{FUNCTION (NORMALISED)}}}$$

(Integrate) $$f(r) = \left(\frac{I_0}{2\pi\sigma^2}\right)\int_0^{2\pi} d\phi \int_0^{\pi} a^2 \sin\theta\, d\theta\; e^{-(r^2+a^2-2ar\cos\theta)/2\sigma^2}$$

Substitute $x = \cos\theta$, $\theta = \pi \rightarrow x = -1$

$dx = -\sin\theta\, d\theta$, $\theta = 0 \rightarrow x = 1$

(Substitute) $$\left(\frac{1}{I_0}\right) f(r) = \left(\frac{a}{\sigma}\right)^2 \int_{-1}^{1} e^{-(r^2+a^2)/2\sigma^2}\, e^{+2arx/2\sigma^2}\, dx$$

(Separate) $$= \left(\frac{a}{\sigma}\right)^2 e^{-(r^2+a^2)/2\sigma^2} \int_{-1}^{1} e^{2arx/2\sigma^2}\, dx$$

(Integrate exponential) $$= \left(\frac{a\sigma^2}{r\sigma^2}\right) \cdot e^{-(r^2+a^2)/2\sigma^2} \cdot \left[e^{2ar/2\sigma^2} - e^{-2ar/2\sigma^2} \right]$$

(Difference of two squares →) $$f(r) = \left(\frac{a I_0}{r}\right)\left[e^{-(r-a)^2/2\sigma^2} - e^{-(r+a)^2/2\sigma^2} \right]$$

Supporting Information 4

Sample image data from this study is uploaded at data.cam.ac.uk/

LINK: https://www.repository.cam.ac.uk/handle/1810/251135
