# Supplementary material for: Ellipsoid Localization Microscopy Infers the Size and Order of Protein Layers in Bacillus Spore Coats
Source: Biophys J. 2015 Nov 17;109(10):2058–66. doi: 10.1016/j.bpj.2015.09.023 (PMC4656864; doi:10.1016/j.bpj.2015.09.023)
Supplement: Document S2. Article plus Supporting Material [file mmc2.pdf]

Article

Ellipsoid Localization Microscopy Infers the Size and Order of Protein Layers in *Bacillus* Spore Coats

Julia Manetsberger,[1] James D. Manton,[1] Miklos J. Erdelyi,[2] Henry Lin,[1] David Rees,[3] Graham Christie,[1] and Eric J. Rees[1,*]

[1]Department of Chemical Engineering and Biotechnology, University of Cambridge, Cambridge, United Kingdom; [2]Department of Optics and Quantum Electronics, University of Szeged, Szeged, Hungary; and [3]Malvern, Worcestershire, United Kingdom

ABSTRACT Multilayered protein coats are crucial to the dormancy, robustness, and germination of bacterial spores. In *Bacillus subtilis* spores, the coat contains over 70 distinct proteins. Identifying which proteins reside in each layer may provide insight into their distinct functions. We present image analysis methods that determine the order and geometry of concentric protein layers by fitting a model description for a spheroidal fluorescent shell image to optical micrographs of spores incorporating fluorescent fusion proteins. The radius of a spherical protein shell can be determined with <10 nm error by fitting an equation to widefield fluorescence micrographs. Ellipsoidal shell axes can be fitted with comparable precision. The layer orders inferred for *B. subtilis* and *B. megaterium* are consistent with measurements in the literature. The aspect ratio of elongated spores and the tendency of some proteins to localize near their poles can be quantified, enabling measurement of structural anisotropy.

INTRODUCTION

The remarkable ruggedness of bacterial spores of the orders *Bacillales* and *Clostridiales* is conferred upon these microorganisms in part by their proteinaceous coats.

In *Bacillus subtilis*, elegant studies have established that the spore has an elongated spheroidal shape and that its outer portion is a multilayered coat (1). Electron and fluorescence microscopy experiments support the subdivision of the coat into defined basement, inner, outer, and crust layers, at least in *B. subtilis*, whereas spores of other species, including *B. cereus* and *B. megaterium*, have a morphologically distinct outermost layer called the exosporium (Fig. 1) (2,3). Some important coat layers have a thickness of <50 nm; cross-sectional analysis of fluorescence micrographs has identified the layers in which some coat proteins reside, but diffraction-limited resolution means that peak-finding methods cannot determine the exact diameter or thickness of these layers (4,5). A more precise method would be useful to elucidate the distribution and relationship of proteins within each layer.

Many bacterial spores have approximately spherical or ellipsoidal shapes, and these simple geometries are amenable to analysis by model-fitting methods (6). In a recent study on the distribution of proteins that make up the tegument of spherical HSV-1 virus particles, the characteristic diameters of distinct protein layers were precisely determined by fitting the parameters of a model shell structure to the observed superresolution images (7). In this article, we present a model-fitting approach to identify the order of protein layers in *B. subtilis* and *B. megaterium* spore coats from widefield fluorescence microscopy data. We first establish an algebraic model for the microscopy image expected from a thin fluorescent spherical shell, such as a fluorescent fusion protein incorporated in a coat (Fig. 2). This model can be fitted to observed images to precisely infer the diameter of different protein layers in near-spherical spores such as *B. megaterium* QM B1551. We then extend the method by fitting Monte Carlo models for spherical and ellipsoidal fluorescent shells, which make it possible to estimate the mechanical anisotropy of *B. subtilis* coats in addition to their protein-layer order. Finally, the inferred spore parameters can be fed back into the image model to generate a superresolved reconstruction of either an individual or an average spore.

MATERIALS AND METHODS

Bacterial strains and preparation of spores

Bacillus strains, listed in Table S1 in the Supporting Material, were cultured on Luria-Bertani agar or broth at 30°C, supplemented with antibiotics (5 μg/mL chloramphenicol or 1 μg/mL erythromycin and 25 μg/mL lincomycin) where appropriate. Spores were prepared by nutrient exhaustion using 2× Schaeffer's glucose medium (*B. subtilis*) (8) or supplemented nutrient broth (*B. megaterium*) (9), purified from cellular debris by several cycles of centrifugation and resuspension in ice cold deionized water, and then stored as suspensions ($OD_{600} = 50$) in water at 4°C. *Escherichia coli* DH5α, employed for molecular cloning and plasmid propagation procedures, was cultured on LB medium supplemented with carbenicillin (50 μg/mL).

Submitted July 9, 2015, and accepted for publication September 21, 2015.

*Correspondence: ejr36@cam.ac.uk

This is an open access article under the CC BY license (http://creativecommons.org/licenses/by/4.0/).

Editor: Paul Wiseman.

© 2015 The Authors

0006-3495/15/11/2058/9

http://dx.doi.org/10.1016/j.bpj.2015.09.023

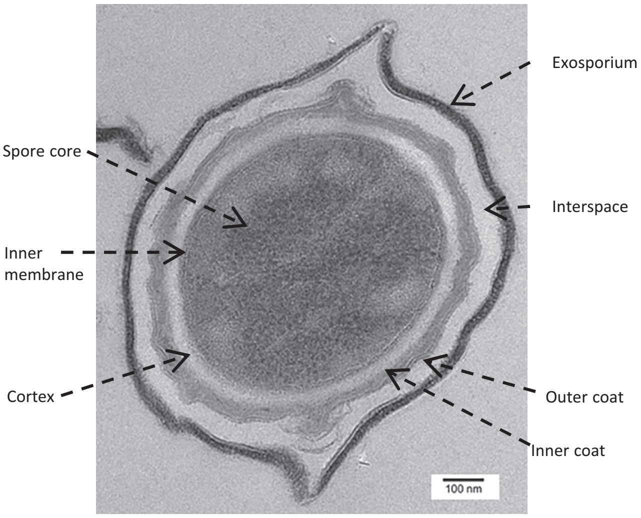

FIGURE 1 Thin-section transmission electron micrograph showing the layers of the coat in *B. megaterium* QM B1551.

Construction of spores with fluorescent fusion proteins

The construction of *B. megaterium* strains bearing C-terminal green fluorescent protein (GFP) fusions to SleL (BMQ_0021), BMQ_4051, BMQ_3035, and BMQ_0737 proteins has been described previously (10). Strains with GFP fusions to CotE (BMQ_4110) and CotW (BMQ_pBM60030) were prepared similarly. GFP fusions to putative coat proteins CotX1 (BMQ_pBM60028) and CotX2 (BMQ_pBM60029) were prepared by polymerase chain reaction (PCR) amplification of the respective open reading frames (minus stop codons) plus upstream regulatory sequences, and then using Gibson assembly (New England Biolabs, Ipswich, MA) to fuse the respective genes in frame with *gfp* located on the linearized low-copy episomal plasmid pHT315. The resultant plasmids were isolated from transformant *E. coli* and introduced to *B. megaterium* QM B1551 by polyethylene-glycol-mediated transformation, and spores were prepared as described above. *B. subtilis* SleL-GFP, CotG-GFP, and CotZ-GFP strains were prepared by cloning the appropriate gene of interest-*gfp* PCR amplicon into plasmid pDG1662, including upstream regulatory sequences. Transformant *B. subtilis* strains that had undergone double homologous recombination at the nonessential *amyE* locus on the chromosome were identified on starch-containing medium, verified by PCR, and then sporulated as described above.

Microscopy

Three microliters of the prepared spore suspension was dispersed onto a flat agar pad on a microscope slide, and sealed under a coverslip. The samples were imaged on an Olympus (Center Valley, PA) BX53 microscope with a 100× 1.30 NA oil objective lens, illumination from a mercury lamp, filters for GFP fluorescence, and a Retiga 2000R CCD camera (QImaging, Surrey, British Columbia, Canada), giving a pixel width of 74 nm on the specimen and 12-bit gray levels. Frames of image data were recorded as 1600 × 1200-pixel Tiffs.

Image simulation

Simulated fluorescence images of spherical spores were generated by TestSTORM, a set of Matlab software for validating localization microscopy protocols (11). Well-separated spherical shells of radius exactly 500 nm were defined with their centers in the focal plane of a widefield microscope. Ten thousand fluorescent molecules were randomly placed on the surface of each shell to simulate homogenous labeling. Frames of image data were simulated by summation of fluorescence using a realistic three-dimensional point-spread function (PSF) including the effects of defocus, for an oil objective lens of 1.3 NA. The fluorescence emission wavelength was defined to be 500 nm, and the image data were collected on square pixels of width 74 nm with a 100% fill factor.

Image analysis

A typical micrograph in this study exhibits ~200 spores, distributed sparsely over a 120 μm × 90 μm area with a dark background. These raw data were analyzed by a segmentation, fitting, and quality-control method (Fig. 3). Matlab scripts developed for this purpose are given in Section S2 of the Supporting Material. The data were first segmented; a circular Hough transform estimated the positions of spores. Typically, all the individual spore images were identified by this step, including elongated spores, together with some false positives from fragments of fluorescent matter and clumps of spores. An anticollision filter was applied to prevent double-counting of the elongated *B. subtilis* spores, which sometimes registered as two nearby circles. The shell-image models were fitted to raw-image data from 27 × 27-pixel regions centered on every candidate. Matlab's fitnlm function was used to fit algebraic expressions to the image data, and a least-squares iterative random search was developed for the Monte Carlo models. Dark background signal was removed by setting a zero signal level at the median value of all pixels darker than the mean value in the fitting region. A quality control criterion was then used to identify which candidate data contained an image of an individual spore that had been accurately fitted. Candidates were accepted if the fitting process yielded a plausible value of σ, with the accepted range being any positive value up to twice the size of the known microscope PSF radius. This simple criterion was found to be accurate for accepting individual spore images and rejecting overlapping and fragmented spores.

For each field of image data, the unweighted average of all the shell radii fitted to accepted candidates was evaluated for the spherical spore models and presented in Fig. 4. For ellipsoidal models, the semiaxis lengths (a and b) were fitted, but to facilitate comparison with the spherical model, they are presented as the radius of a sphere of equal volume, $(a^2b)^{1/3}$, and the aspect ratio (b/a). This average measurement was repeated on five independent fields of spores, and the standard deviation of these observations was used to identify the random error of the method. The residual error of the analysis for each spore was evaluated as the sum of square residuals between fitted pixel values ($I^{\rm fit}$) and data ($I^{\rm data}$).

$$\varepsilon = \frac{\sum_j \left(I_j^{\rm data} - I_j^{fit}\right)^2}{\sum_j \left(I_j^{\rm data}\right)^2}. \tag{1}$$

RESULTS

Algebraic model for the image of a spherical fluorescent protein layer

The captured image in an ideal optical microscope can be calculated as the convolution of the object function with the PSF of the instrument (12). In a fluorescence microscope, the positions of excited fluorescent molecules determine the object function, and nonexcited molecules do not contribute to the final image. Our model for a fluorescent shell under uniform widefield illumination is shown in Fig. 2 and in Section S3 of the Supporting Material. The

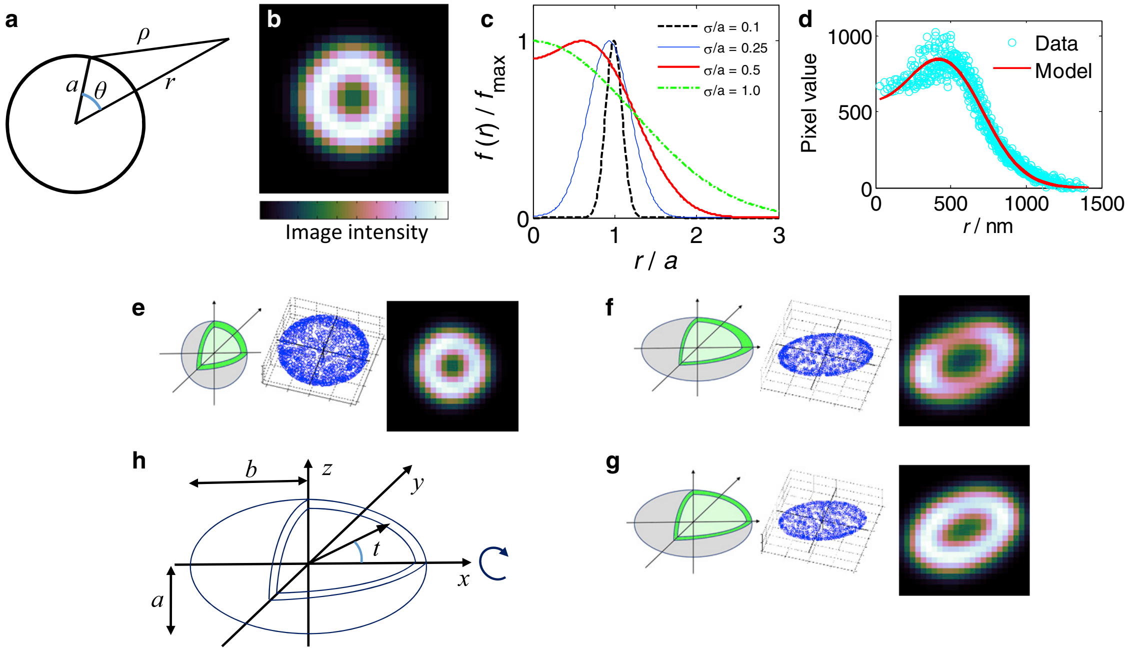

FIGURE 2 Models for the microscopy images of spheroidal protein shells. (*a*) The geometry of a spherical fluorescent shell of radius a can be used to establish an equation for the radial intensity distribution of its fluorescence image, $f(r)$. (*b*) A sample image generated by $f(r)$. (*c*) A more general plot detailing how $f(r)$ varies with the radius of the microscope PSF. Note that the contour of maximum image brightness lies inside the shell radius, due to blurring by the microscope. The cubehelix color map, which is linear in perceived brightness and maximally distinct in color, is ideal for visualizing the function (26). (*d*) A typical, real fluorescence image of *B. megaterium* can be very effectively fitted by $f(r)$. (*e–g*) A Monte Carlo model of a spherical fluorescent coat layer generates images similar to those generated by $f(r)$, and can be adapted to simulate elongated spores either by stretching the sphere into an ellipsoid, which results in relatively denser fluorescent molecules at the poles (*f*) or sampling an ellipsoidal shell with uniform fluorescent labeling density (*g*). Real images of elongated spores often lie between cases (*f*) and (*g*). (*h*) Coordinate system for the ellipsoidal model. To see this figure in color, go online.

object function is a uniformly bright thin spherical shell of radius a centered at Cartesian coordinates (x_0, y_0) in the object plane ($z = 0$). A spherically symmetric Gaussian, $h(\rho)$, is used to model the PSF of an aberration-free microscope, which is a good approximation for a confocal microscope and a popular simplification for widefield systems (13,14). $h(\rho)$ describes the radial intensity distribution detected in the image of a point source of intensity I, at a displacement from the geometric image center corresponding to a distance ρ in the object frame. (Here, a, ρ, and the PSF radius, σ, are all stated in the length scale of the object frame.)

$$h(\rho) = \left(I/2\pi\sigma^2\right)e^{\left(-\rho^2/2\sigma^2\right)} \tag{2}$$

The fluorescence intensity, $f(r)$, in the image of a spherical shell, at a point corresponding to a radial distance in the object frame, $r = ((x - x_0)^2 + (y - y_0)^2)^{1/2}$, from the center of the sphere, is determined by integrating the contribution from all parts of the fluorescent surface. The image intensity is therefore given by Eq. 3, where I_0 is the fluorescence emission per unit area of the shell and (θ, ϕ) are spherical polar coordinates of a point on its surface.

$$f(r) = \frac{I_0}{2\pi\sigma^2}\int_0^{2\pi} \mathrm{d}\phi \int_0^{\pi} a^2 \sin(\theta) e^{-\left(\frac{r^2+a^2-2ar\cos(\theta)}{2\sigma^2}\right)} \mathrm{d}\theta \tag{3}$$

Integrating via the substitution $v = \cos(\theta)$, it follows that the fluorescence image of the thin spherical shell has a remarkably simple radial intensity distribution.

$$f(r) = aI_0\left(e^{-(r-a)^2/2\sigma^2} - e^{-(r+a)^2/2\sigma^2}\right)\Big/ r \tag{4}$$

The diameter of a thin spherical fluorescent shell can be quantified by fitting the parameters of Eq. 4 to its fluorescence microscopy image. Assuming the microscope has a finite PSF ($\sigma > 0$), $f(r)$ is well behaved for fitting purposes apart from a singular point at $r = 0$, which is removable, because $f(r)$ has a finite value in the limit $r \rightarrow 0$. By L'Hôpital's rule, or directly as $4\pi a^2 h(a)$,

$$\lim_{r\rightarrow 0} f(r) = \frac{2a^2 I_0}{\sigma^2} e^{-a^2/2\sigma^2}. \tag{5}$$

Five parameters of a spherical shell must be identified from its image data: its center position (x_0, y_0), radius a, and uniform brightness per unit area I_0, as well as the instrumental blurring radius, σ. The process of identifying and fitting an image model to microscopy data is illustrated in Fig. 3. Fig. 4 presents the dimensions that are thereby inferred for *B. megaterium*, *B. subtilis*, and calibration image data that were simulated using a physically realistic PSF and a set of spherical shell specimens with a ground-truth radius of 500 nm.

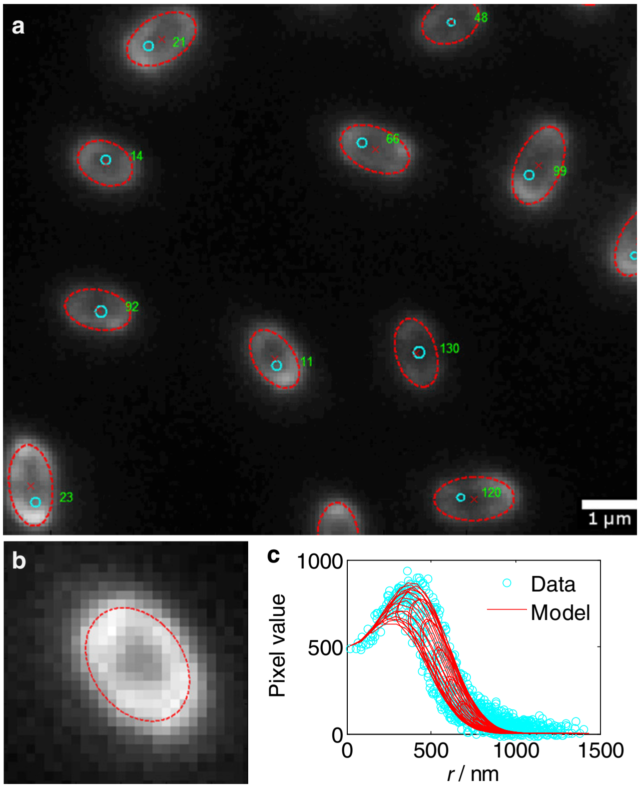

FIGURE 3 Computational segmentation and fitting can determine the geometry of ellipsoidal spores. (*a*) Fluorescence images of *B. subtilis* are approximately located by a circle-finding algorithm (*blue circles*) and assigned a number for indexing. (*b*) Each candidate image is used to fit the parameters of an ellipsoidal image model, with the shell outline and center shown in red over the gray image data. (*c*) The pixel values in a typical image of *B. megaterium* can be effectively fitted by the ellipsoidal model.

In a real spore, the shape of each protein layer in the coat is likely to deviate from the scenario of a perfectly spherical and uniformly bright shell, and the layers will have finite thickness. When Eq. 4 is fitted to image data collected from real spores, the apparent value of σ tends to increase, because it contains a contribution from the structure of the specimen, as well as the PSF of the microscope. Furthermore, the value of a fitted to an aspherical specimen is not a definitive physical parameter; however, a is still closely related to the average shell radius of nearly spherical specimens and may be an accurate enough approximation to identify details such as the order of concentric protein layers.

For a shell of fluorescent protein that is spherical with finite thickness, $f(r)$ could be integrated over a to predict the radial distribution of fluorescence intensity in its image, $f_2(r)$.

$$f_2(r) = \int_{a'-d}^{a'+d} f(r)\mathrm{d}a = \left(\frac{I_0\sigma}{2r}\right)\left[\sqrt{2\pi}r\,\mathrm{erf}\left(\frac{a-r}{\sqrt{2}\sigma}\right) + \sqrt{2\pi}r\,\mathrm{erf}\left(\frac{a+r}{\sqrt{2}\sigma}\right) + 2\sigma\left(e^{-(a+r)^2/2\sigma^2} - e^{-(a-r)^2/2\sigma^2}\right)\right]_{a'-d}^{a'+d} \tag{6}$$

Here, the shell has radius a' to its midpoint, thickness $2d$, and the error function is denoted erf. The main difficulty in using $f_2(r)$ to infer fluorescent layer thickness is that the instrumental blurring radius is ambiguous with the shell thickness. Visually, the image modeled by $f_2(r)$ appears more blurred if σ increases, and the effect of increasing d is similar. In practice, it is difficult to accurately fit d from image data unless $\sigma << d$. An accurate result can be obtained if σ is independently known by calibration; however, in this case there is a risk that an aspherical or inhomogenously fluorescent specimen may induce significant errors into an inferred value of the shell thickness.

Monte Carlo models for shell images

Alternatively, the image of a spherical shell can be modeled by the Monte Carlo method shown in Fig. 2 *e*. The image is simulated by summing contributions from many (typically 2500) fluorophores that are randomly placed on the surface of a sphere. The model parameters are essentially the same as in the algebraic model: the sphere center position, (x_0, y_0); radius, a; uniform brightness per fluorophore I; and radius of blurring of the image, σ, attributed to the microscope PSF. We use the trig method for picking random points uniformly on a spherical shell (15). For each point, we generate the azimuthal coordinate, ϕ, randomly from a uniform distribution on $[0, 2\pi]$, and by picking u from a uniform distribution on $[-1, 1]$, we establish the polar coordinate as $\cos(\theta) = u$. The Cartesian coordinates of the point are given by $x = a\sin(\theta)\cos(\phi)$, $y = a\sin(\theta)\sin(\phi)$, and $z = a\cos(\theta)$. This ensures that the probability, $\iint \mathrm{d}u\mathrm{d}\phi/4\pi = \iint \sin(\theta)\mathrm{d}\theta\mathrm{d}\phi/4\pi$, of choosing a point in a region of the shell of area $\mathrm{d}S = a^2\sin(\theta)\mathrm{d}\theta\mathrm{d}\phi$ is proportional to the area and independent of position. The contribution of each fluorophore to each pixel value in the recorded image is evaluated by Eq. 2. This method produces images identical to those produced by Eq. 4 provided enough fluorophores are simulated; and although the Monte Carlo approach is computationally much slower, it is easier to adapt for more complex specimens such as elongated shells or different microscope PSFs.

Monte Carlo models for the image of an ellipsoidal fluorescent shell are shown in Fig. 2, *f–g*. We consider only a prolate ellipsoid of revolution with its long axis lying flat in the object plane, because this is a simple case that resembles our images of elongated *B. subtilis* spores spread over a coverslip. Only two new parameters are needed in this scenario—the aspect ratio of the ellipsoid (b/a) and its orientation. For a prolate ellipsoid with its major axis aligned on the x axis, we use a as the semiminor axis length, and the surface is defined by

$$F(x, y, z) = \frac{x^2}{b^2} + \frac{y^2}{a^2} + \frac{z^2}{a^2} = 1. \tag{7}$$

In Fig. 2 *f*, the image of a stretched sphere is obtained by simulating points (x', y', z') on a unit sphere using the trig

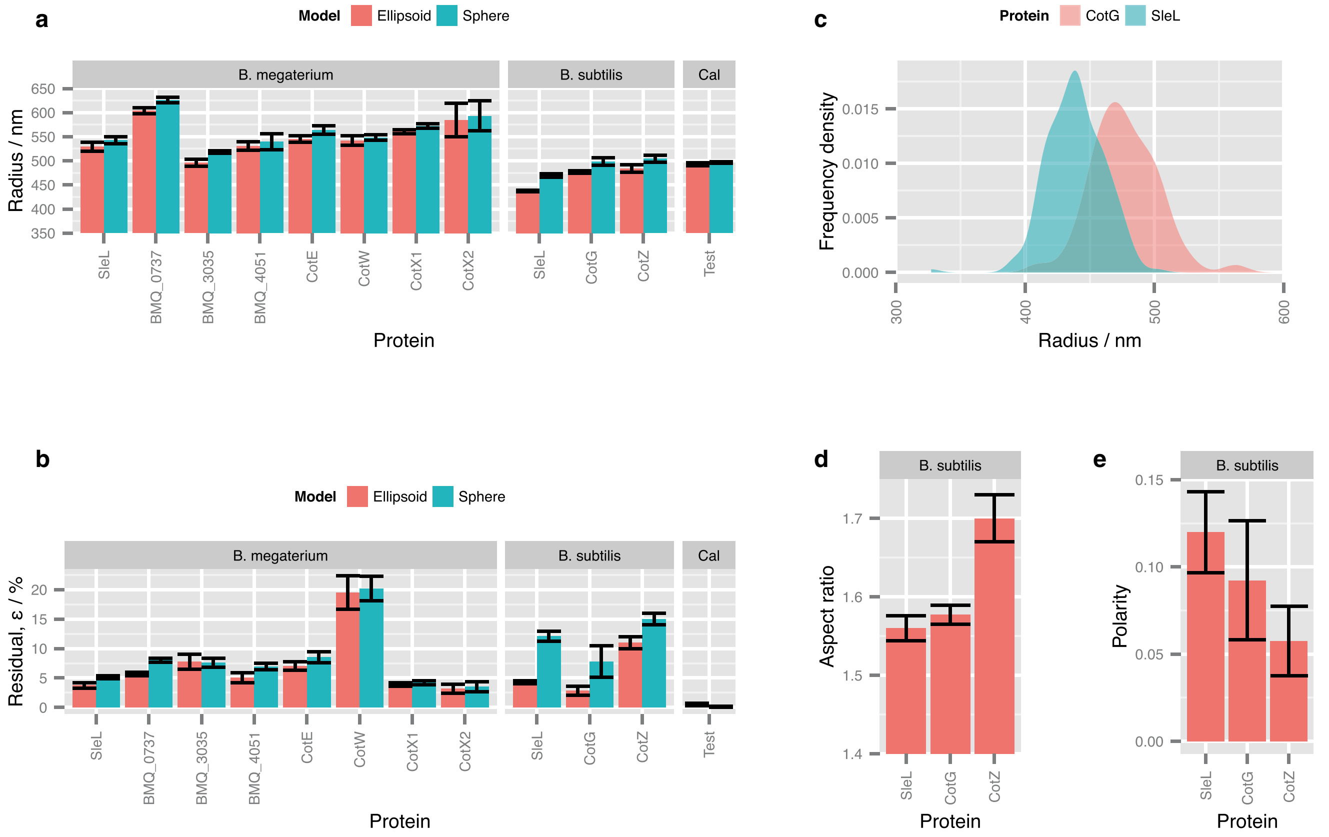

FIGURE 4 Model-fitting estimates of a fluorescent protein layer radius in *B. megaterium* and *B. subtilis*, and in simulated calibration data with a ground-truth radius of 500 nm (*Cal*). (*a*) The average layer radius was obtained by analyzing at least three separate fields of typically 200 spores each, and the error bars indicate the standard deviation of these measurements. For the ellipsoidal models, the radius of a sphere of equal volume is presented. (*b*) Residual errors of fitted models. (*c*) The distribution of radii fitted to SleL and CotG in 200 individual *B. subtilis* spores. (*d*) The aspect ratio of ellipsoidal *B. subtilis* spores. (*e*) The polarity parameter quantifying the tendency of proteins to localize preferentially at the spore poles. To see this figure in color, go online.

method, then applying a linear stretch to obtain $x = bx'$, $y = ay'$, and $z = az'$, and finally a rotation about the z axis. Just as with the spherical model, the image is then generated by summing contributions from a fluorophore at each point using Eq. 2. An essential feature of the stretched-sphere model is that the fluorophores near its poles are denser, when measured per unit surface area of the ellipsoid, than the ones near its equator due to the anisotropic stretch. Therefore, the image of the stretched sphere has relatively bright poles compared to the rest of its image. This is noteworthy, because fluorescence images of *B. subtilis* coats often resemble such a pattern. It is thought that the protein layers initially form at the poles and accumulate there more thickly (1). A conceivable alternative is that some protein layers might be thicker at the poles if they initially form as uniform spherical shells, which are subsequently deformed (by an axial stretch or a cylindrical contraction) in a manner similar to the stretched-sphere image simulation model. This latter case seems less likely, but it is interesting to note that an image model comparison could provide evidence to distinguish these cases.

A second Monte Carlo model (Fig. 2 *g*) generates the image of an ellipsoidal shell that is fluorescently labeled with equal density on each unit area of its surface, which we call a uniform ellipsoid. This might correspond to a protein layer assembled at constant thickness on an elongated forespore without any subsequent mechanical deformation. The uniform ellipsoid model simulates fluorophores on a shell in the same way as the stretched-sphere model, and then randomly discards some points with a probability of retention ($p = g/g_{\max}$) proportional to the local area scale factor, g, that acts when the ellipsoid surface is produced from the unit sphere. This is an established method for picking points uniformly on an ellipsoid surface (15,16).

$$p = \frac{g}{g_{\max}} = a\left(\frac{x^2}{b^4} + \frac{y^2}{a^4} + \frac{z^2}{a^4}\right)^{1/2} \tag{8}$$

The resulting images have a far more uniform contour of maximum brightness than in the stretched-sphere case, although the poles are still slightly brighter than the equator due to the greater amount of curved fluorescent surface near the focal plane.

Experimental images of *B. subtilis* with GFP-tagged coat proteins tend to exhibit brighter fluorescence at the poles, but the extent of the bias seems to vary between proteins

and individual specimens. Therefore, a general polarized ellipsoid model is justified for characterizing these images. To quantify the apparent degree of preferential localization at the spore poles, we arbitrarily introduce a biased retention probability, p_b, to the uniform ellipsoid model. Here, $\sin(t) = (y^2 + z^2)^{1/2}/(x^2 + y^2 + z^2)^{1/2}$ is zero at the spore poles and increases smoothly to unity at the equator, so a positive value of the parameter q (which we call the polarity) corresponds to a fluorophore localized more densely at the poles and a negative value to one found preferentially at the equator. The normalization term C is 1 for $q \geq 0$ and $(1 - q)^{-1}$ for $q < 0$.

$$p_b = C\left(\frac{g}{g_{max}}\right)(1 - q\sin(t)) \tag{9}$$

By fitting the polarized ellipsoid model to image data, it may be possible to estimate whether any measured difference between the fluorescence density at the spore poles and equator is consistent with the stretched-sphere case, or some other mechanism of uniform or nonuniform layer construction around an elongated core.

DISCUSSION

The algebraic model for a spherical shell image is based on an approximate PSF, so it was crucial to validate the inference method using calibration data for specimens of exactly known geometry. In this case, we simulated fluorescence images for perfectly spherical specimens of radius 500 nm using separate software that implements a realistic imaging description (11). Our inference method recovered a radius of 497 ± 2 nm for these specimens, which shows that the systematic bias arising from mathematical approximations is small. In fact, this bias can almost be neglected during this study, because the repeatability in the average radius of a specific coat-protein layer between batches of spores was at best 6 nm. This finite random uncertainty probably arises from natural variation or imperfectly spheroidal shape or inhomogeneous fluorescence of spores, and it might be improved by analyzing large numbers of more brightly fluorescent spores, but it is already good enough to distinguish protein layers. Of course, the aspherical and inhomogeneous nature of coats might also systematically bias the inferred radius, although given the accurate fitting of the image data by the models, such a bias is probably small. The very small bias of the algebraic model meant that the simple spherical Gaussian PSF was also used in the Monte Carlo image models to facilitate comparison of results, since there was little to be gained by implementing a more sophisticated case.

Intriguingly, the equation $f(r)$ for the radial intensity distribution in a spherical spore image explains the observation of Imamura that when the diameters of protein layers in a *B. subtilis* coat were inferred by measuring the separation between peaks of fluorescence intensity in image cross sections, a diameter ~60 nm bigger was identified with GFP fusions than with RFP (4). This result can be understood from Fig. 2 *c*: the peak of $f(r)$ is equivalent to the contour of maximum brightness in an image of a spherical shell. The radial position of this contour is biased inward from the true shell radius (a) by an offset that increases when the blurring of the instrument (σ) becomes more significant. Therefore, suppose that Imamura's *B. subtilis* image data were measured from (approximately spherical) spores with a fluorescent layer radius of 600 nm, and the instrument resolution is $1.22\lambda/\mathrm{NA}_{total}$, with $\lambda_{RFP} \approx 600$ nm, $\lambda_{GFP} \approx 500$ nm, $\mathrm{NA}_{objective}$ 1.4, $\mathrm{NA}_{condenser}$ 0.9, and a PSF radius approximately one-half of the resolution, so $\sigma_{RFP} \approx 160$ nm and $\sigma_{GFP} \approx 130$ nm. In this case, calculation shows that the peak of $f(r)$ is at a radial position of 550 nm for RFP and 570 nm for GFP, predicting a difference of ~40 nm in the inferred diameter. This broadly explains Imamura's admirably precise results. The increasing positional offset that affects the contour of maximum brightness as σ/a increases also means that attempts to infer coat layer thickness by measuring the displacement of peak image brightness between proteins in consecutive layers (which have different radius a) are slightly biased even if the same fluorescent color (affecting σ) is used.

Studying *B. megaterium* SleL-GFP spores by fitting the spherical image model quickly establishes three conclusions. First, the spherical-spore model accurately matches the observed images with only a small residual error, $\varepsilon \approx 5\%$, which suggests that this model is realistic enough to support robust inferences of *B. megaterium* structure. Second, the population of spores in one entire frame of data has a distribution of inferred SleL-GFP sphere radii with a standard deviation of ~50 nm, probably due to varying quantities of material in each spore core. However, the mean radius of SleL-GFP in an entire frame of raw data (~200 spores) is precisely repeatable, with a standard deviation of <10 nm when several frames of data are compared. A repeatability finer than 10 nm was also obtained for the mean radius of BMQ_0737-GFP and CotX1-GFP, both of which emitted bright fluorescence and produced high-contrast image data that supported precise estimates of the shell size. The poorest repeatability of inferred sphere radius was for CotX2-GFP, with a standard deviation of 26 nm, apparently due to the low brightness of this specimen, which worsened both the precision of parameter fitting and the number of identifiable spores. Third, the average spherical radii inferred for the putative inner-coat proteins in *B. megaterium* (SleL, BMQ_3035, and BMQ_4051) were smaller in every case than the values fitted to the outer-coat proteins (CotW, BMQ_0737, and both CotX homologs), typically by a difference of 50 nm. Therefore, knowing the typical radii of the inner and outer coats, the inference was precise enough to establish which layer contained a particular protein.

The radius of the CotE protein layer is of especial interest, because CotE has a morphogenetic role in attaching the outer coat to the inner coat of *B. subtilis* (17,18). The fluorescent shell model-fitting analysis inferred that CotE-GFP is localized exactly between the inner- and outer-coat proteins in *B. megaterium*. This result suggests that CotE may play a similar morphogenetic role in *B. megaterium*. Importantly, the fluorescent shell analysis was precise enough to provide evidence for the role of CotE in *B. megaterium*, and could even be used to study morphogenetic proteins in less well characterized species. In addition, the inferred radii of SleL, CotE, and CotW proteins in *B. megaterium* lie significantly inside the other putative outer-coat and exosporium proteins. This is consistent with the observation that the proteins BMQ_0737, CotX1, and CotX2 bind anti-GFP antisera strongly in immunolabeling assays (10), which implies that those are the outermost proteins, whereas no such signal was seen with SleL, CotE, and CotW.

Accurate characterization of *B. subtilis* coat layers, unlike the nearly spherical *B. megaterium* spores, required an ellipsoidal image model. For *B. subtilis*, fitting the polarized ellipsoid model to its image data significantly improved the residual error of the final fit, and therefore, the aspect ratio and polarity inferred by this analysis are essential data. For *B. megaterium*, the simpler model of a uniformly fluorescent spherical shell is preferred, because it fitted the data as closely as the ellipsoid model and has fewer free parameters. Therefore, the ellipsoidal parameters that could be inferred for *B. megaterium* should not be considered and are not presented.

Sets of *B. subtilis* images were obtained, and the average axis lengths inferred by fitting the ellipsoidal model were repeatable, with standard deviations typically more precise than 10 nm. The order of protein layers was therefore identifiable, and the result was exactly as expected from the literature, i.e., the cortex lytic enzyme SleL (also known as YaaH) is a known inner-coat protein, whereas CotG and CotZ are components of the outermost crust (5,19).

The ability to measure the exact dimensions of *B. subtilis* coat layers is useful not only for establishing layer order, but also in biophysical studies. For example, the coat of *B. subtilis* cracks open during germination, and the crack typically opens around the equator of the spore and not along its long axis. It is likely that the architecture of the coat is weaker to crack growth around the equatorial direction than in the axial direction, perhaps due to thinner protein layers at the equator or to anisotropic strengthening of the underlying cell wall by helical glycan strands (20). The exact dimensions of a coat can be used to evaluate its surface tension in different directions: for a thin-walled ellipsoidal pressure vessel containing an internal turgor pressure, Kuznetsov derived a model for the anisotropic surface tension (21). For an aspect ratio *b*/*a*, it was found that the surface tension anisotropy in the structure was as follows:

$$\frac{\gamma_y}{\gamma_x\left(\theta = \frac{\pi}{2}\right)} = \frac{4(b/a)^2}{3(b/a)^2 + 1}, \tag{10}$$

where γ_y is the surface tension in the direction around the equator of the shell, and $\gamma_x(\theta = \pi/2)$ is the surface tension at the equator in the direction parallel to its long axis. For the aspect ratio fitted to the SleL protein layer in *B. subtilis*, the surface tension anisotropy given by Eq. 10 is ~1.2. If the shell of the spore were mechanically isotropic, it might be expected to crack open along a direction perpendicular to the largest stress—i.e., it would crack along its long axis, since γ_y is the larger surface tension. The fact that it does not places a lower bound on the anisotropic mechanical reinforcement of the spore shell.

The polarity parameter inferred for different proteins in *B. subtilis* suggests that SleL is located most preferentially at the spore poles, and that the protein layers located farther toward the outside of the coat, CotG and then CotZ, are increasingly more uniform in distribution. Given that the spore shell seems to possess mechanical anisotropy, it would be interesting to identify a protein that might be involved in mechanical reinforcement. Perhaps such a protein might be localized at the spore equator, but none of the ones we studied were.

In Fig. 5 *a*, a reconstructed superresolution image of ~50 individual *B. subtilis* spores was produced by feeding the structural parameters inferred from their image data back into the image model. In this case, both the inferred spore parameters and the reconstructed images were obtained with the polarized ellipsoid model, which captured the most information about *B. subtilis* structure. When generating reconstructed images of thin shells using a mathematical model, the PSF parameter can be made arbitrarily small. In practice, we set the PSF radius to half the estimated fluorescent layer thickness (i.e., 25 nm), to relate the reconstructed image to the fluorescence density of the spore. As well as reconstructing super-resolved images of individual spores, the average structural parameters of spore populations can be combined to generate a reconstructed visualization of an average spore structure (Fig. 5, *b* and *c*). This reconstruction can be used to visualize the layer order, aspect ratio, and polarity of different protein layers. Of course, these reconstructed images are merely inferences based on special assumptions (such as the spores being exactly ellipsoidal). Although the reconstructions can be useful as a visualization of the fitted dimensions (and for comparison with the raw data), they must not simply be interpreted as an unbiased image captured by a high-resolution instrument. In practice, the reconstructions should be accurate, provided that the image data contain nonoverlapping, in-focus images of structures each of which comprises a single, bright fluorescent layer that can be accurately represented as a spheroid. Overlapping or adjacent images tend to severely bias the parameters inferred by fitting the single

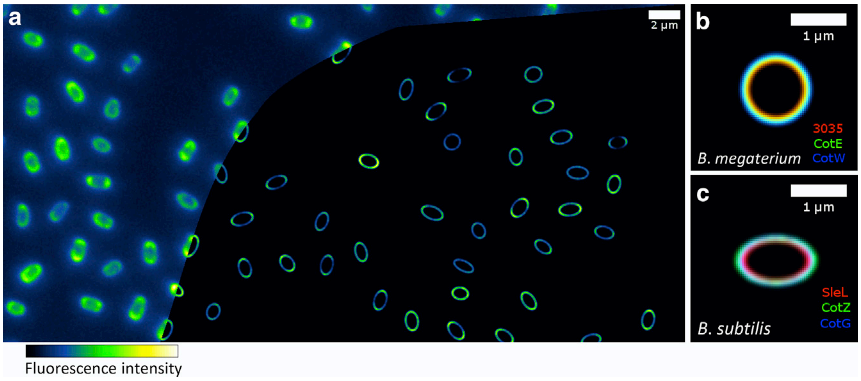

FIGURE 5 Superresolved reconstruction of fluorescence intensity in *B. subtilis* spores. (*a*) The parameters fitted to SleL-GFP *B. subtilis* images are fed back into the ellipsoid model for its image, while decreasing the inferred PSF to remove instrumental blurring. The original widefield image data are shown to the left of the reconstruction for comparison. (*b* and *c*) The inferred parameters of multiple protein layers are averaged and visualized in the same way to illustrate the structural details captured by model fitting for *B. megaterium* (*b*) and also for *B. subtilis* (*c*). Scale bars, 2 μm (*a*) and 1 μm (*b* and *c*).

fluorescent layer model, and these cases should be discarded by anticollision filtering or by thresholding the fitted model to accept a trusted range of parameters (fitted PSF radii tend to be increased when the model fits data poorly). Importantly, this fluorescent shell analysis method is designed for single fluorescent layers. Our model assumes that there is negligible fluorescent material located away from this single layer, so if there is a small amount of fluorescent protein located elsewhere (which may play an important role in spore morphogenesis), then the model-fitting method will not identify its presence or absence except as a small increase in the residual error of the model. Traces of separate protein may also bias the inferred shell radius, but the bias may be negligible provided that the brightness of the fluorescent layer is high and dominates over separate traces of protein.

The principle of reconstructing an image mathematically at high resolution based on positions (and other structural parameters) inferred from image data is similar to the approach employed in single-molecule localization microscopy (22–25), but our assumptions here are very different. Instead of assuming that photoswitching fluorescence can be used to isolate individual molecule images, we employ an assumption that the observed specimens are elementary ellipsoidal shells with constant fluorescence.

CONCLUSIONS

We have presented models for the images of spheroidal fluorescent shells using both exact algebra and Monte Carlo methods. By computationally fitting these models to fluorescence micrographs of *Bacillus* spores incorporating a fluorescent coat protein, the protein layer radius can be determined with <10 nm precision. This inference is accurate enough to identify whether the protein localizes to the inner coat or the outer coat (and perhaps exosporium), and also to confirm that the morphogenetic CotE protein lies between the inner- and outer-coat layers in *B. megaterium*. These measurements are established by a fairly simple analysis of traditional widefield fluorescence microscopy data, which makes it a practical method for studying specimens in vivo. A superresolved reconstruction of the spore can be obtained by feeding the precise structure parameters back into the image model.

Model fitting to fluorescence micrographs can determine the absolute dimensions of a coat layer, without the bias known to affect peak-fitting methods, and as an optical method it can be applied in vivo. Knowledge of absolute layer dimensions provides insight into the mechanical anisotropy of a spore, and in future, this could enable studies into the anisotropic elasticity of spores by quantifying the axial and lateral rates of expansion in response to turgor pressure during germination. Furthermore, the tendency of some proteins to localize more densely at spore poles can be quantified, which might potentially reveal the state of elastic deformation of a spore, or simply provide information on systematic coat-thickness variation.

This method of ellipsoid localization by model fitting for determining the dimensions of fluorescent shells could be applied to many species of bacterial spores and other approximately ellipsoidal microbes, such as algae, and to microdroplets or vesicles. Even more accurate results may be obtained by fitting exactly the same models to data captured with superresolution instruments.

Software developed in this work, sample image data, and tabulated data on bacterial strains are provided in the Supporting Material.

SUPPORTING MATERIAL

Supporting Materials and Methods and one table are available at http://www.biophysj.org/biophysj/supplemental/S0006-3495(15)00993-5.

AUTHOR CONTRIBUTIONS

J.M. and H.L. prepared and imaged spores. E.J.R. designed the method with H.D.R. and M.E., developed the software with J.D.M., and wrote the article with G.C.

ACKNOWLEDGMENTS

We thank Clemens Kaminski, Romain Laine, and Jose Casas-Finet for inspiring discussions. The authors declare no competing financial interests.

We gratefully acknowledge support from MedImmune through the Beacon collaboration, and from the Engineering and Physical Sciences Research Council Centre for Doctoral Training in Sensor Technologies and Applications (EP/L015889/1).

REFERENCES

1. Driks, A. 1999. *Bacillus subtilis* spore coat. *Microbiol. Mol. Biol. Rev.* 63:1–20.
2. Henriques, A. O., and C. P. Moran, Jr. 2007. Structure, assembly, and function of the spore surface layers. *Annu. Rev. Microbiol.* 61:555–588.
3. McKenney, P. T., A. Driks, and P. Eichenberger. 2013. The *Bacillus subtilis* endospore: assembly and functions of the multilayered coat. *Nat. Rev. Microbiol.* 11:33–44.
4. Imamura, D., R. Kuwana, …, K. Watabe. 2010. Localization of proteins to different layers and regions of *Bacillus subtilis* spore coats. *J. Bacteriol.* 192:518–524.
5. McKenney, P. T., A. Driks, …, P. Eichenberger. 2010. A distance-weighted interaction map reveals a previously uncharacterized layer of the *Bacillus subtilis* spore coat. *Curr. Biol.* 20:934–938.
6. Tarantola, A. 2005. Inverse Problem Theory and Methods for Model Parameter Estimation. SIAM, Philadelphia, PA.
7. Laine, R. F., A. Albecka, …, C. F. Kaminski. 2015. Structural analysis of herpes simplex virus by optical super-resolution imaging. *Nat. Commun.* 6:5980.
8. Nicholson, W. L., and P. Setlow. 1990. Sporulation, germination and outgrowth. *In* Molecular Biological Methods for *Bacillus*. C. R. Harwood and S. M. Cutting, editors. John Wiley and Sons, Chichester, United Kingdom, pp. 391–450.
9. Christie, G., F. I. Ustok, …, C. R. Lowe. 2010. Mutational analysis of *Bacillus megaterium* QM B1551 cortex-lytic enzymes. *J. Bacteriol.* 192:5378–5389.
10. Manetsberger, J., E. A. Hall, and G. Christie. 2014. BMQ_0737 encodes a novel protein crucial to the integrity of the outermost layers of *Bacillus megaterium* QM B1551 spores. *FEMS Microbiol. Lett.* 358:162–169.
11. Sinkó, J., R. Kákonyi, …, M. Erdélyi. 2014. TestSTORM: Simulator for optimizing sample labeling and image acquisition in localization based super-resolution microscopy. *Biomed. Opt. Express.* 5:778–787.
12. Goodman, J. 1996. Introduction to Fourier Optics, 2nd ed. McGraw-Hill, New York.
13. Zhang, B., J. Zerubia, and J. C. Olivo-Marin. 2007. Gaussian approximations of fluorescence microscope point-spread function models. *Appl. Opt.* 46:1819–1829.
14. Stallinga, S., and B. Rieger. 2010. Accuracy of the gaussian point spread function model in 2D localization microscopy. *Opt. Express.* 18:24461–24476.
15. Chen, T., and S. C. Glotzer. 2007. Simulation studies of a phenomenological model for elongated virus capsid formation. *Phys. Rev. E Stat. Nonlin. Soft Matter Phys.* 75:051504.
16. Williamson, J. F. 1987. Random selection of points distributed on curved surfaces. *Phys. Med. Biol.* 32:1311–1319.
17. Zheng, L. B., W. P. Donovan, …, R. Losick. 1988. Gene encoding a morphogenic protein required in the assembly of the outer coat of the *Bacillus subtilis* endospore. *Genes Dev.* 2:1047–1054.
18. de Francesco, M., J. Z. Jacobs, …, P. Eichenberger. 2012. Physical interaction between coat morphogenetic proteins SpoVID and CotE is necessary for spore encasement in *Bacillus subtilis*. *J. Bacteriol.* 194:4941–4950.
19. Imamura, D., R. Kuwana, …, K. Watabe. 2011. Proteins involved in formation of the outermost layer of *Bacillus subtilis* spores. *J. Bacteriol.* 193:4075–4080.
20. Hayhurst, E. J., L. Kailas, …, S. J. Foster. 2008. Cell wall peptidoglycan architecture in *Bacillus subtilis*. *Proc. Natl. Acad. Sci. USA.* 105:14603–14608.
21. Kuznetsov, V. S., A. P. Blinov, …, G. A. Anan’eva. 2007. Surface tension anisotropy in the mechanics of ellipsoidal micelles. *Colloid J.* 69:627–631.
22. Deschout, H., F. Cella Zanacchi, …, K. Braeckmans. 2014. Precisely and accurately localizing single emitters in fluorescence microscopy. *Nat. Methods.* 11:253–266.
23. Rees, E. J., M. Erdelyi, …, C. F. Kaminski. 2013. Elements of image processing in localization microscopy. *J. Opt.* 15:094012.
24. Rees, E. J., M. Erdelyi, …, C. F. Kaminski. 2012. Blind assessment of localisation microscope image resolution. *Opt. Nanoscopy.* 1:12.
25. Cox, S., E. Rosten, …, R. Heintzmann. 2012. Bayesian localization microscopy reveals nanoscale podosome dynamics. *Nat. Methods.* 9:195–200.
26. Green, D. A. 2011. A colour scheme for the display of astronomical intensity images. *Bull. Astron. Soc. India.* 39:289–295.

Biophysical Journal

Supporting Material

Ellipsoid Localization Microscopy Infers the Size and Order of Protein Layers in *Bacillus* Spore Coats

Julia Manetsberger,[1] James D. Manton,[1] Miklos J. Erdelyi,[2] Henry Lin,[1] David Rees, Graham Christie,[1] and Eric J. Rees[1,*]

[1]Department of Chemical Engineering and Biotechnology, University of Cambridge, Cambridge, United Kingdom; and [2]Department of Optics and Quantum Electronics, University of Szeged, Szeged, Hungary

Supporting Information 1

Table S1 Bacterial strains used in this study

**Strain**	**Relevant genotype [a]**	**Source/reference**
B. megaterium strains		
QM B1551	Wild-type strain	P.S. Vary
GFP fusion strains		
JM100	BMQ_0737::pVLG6 (*gfp*) Cm^r	1
JM101	BMQ_3035::pVLG6 (*gfp*) Cm^r	1
JM102	BMQ_4051::pVLG6 (*gfp*) Cm^r	1
JM103	*cotE*::pVLG6 (*gfp*) Cm^r	1
JM104	pHT-*cotX1-gfp* MLS^r	This study
JM105	pHT-*cotX2-gfp* MLS^r	This study
FIU104	*sleL*::pVLG6 (*gfp*) Cm^r	2
B. subtilis strains		
168 1A1	Wild-type strain *TrpC2*	*Bacillus* Genetic Stock Centre
HL100	*TrpC2 amyE::sleL-gfp* Cm^r	This study
HL101	*TrpC2 amyE::cotG-gfp* Cm^r	This study
HL102	*TrpC2 amyE::cotZ-gfp* Cm^r	This study

[a] Abbreviations for antibiotics: Cm^r, chloramphenicol resistance (5 µg/ml); MLSr, lincomycin (25 µg/ml) and erythromycin (1 µg/ml).

<u>References</u>

1. Manetsberger, J., Hall, E. A., and Christie, G. (2014) BMQ_0737 encodes a novel protein crucial to the integrity of the outermost layers of *Bacillus megaterium* QM B1551 spores. *FEMS microbiology letters* **358**, 162-169.

2. Üstok, F. I., Packman, L. C., Lowe, C. R., and Christie, G. (2014) Spore germination mediated by *Bacillus megaterium* QM B1551 SleL and YpeB. *Journal of bacteriology* **196**, 1045-1054.

Supporting Information 2

Image analysis software for the paper:
Ellipsoid localisation microscopy infers the size and order of protein layers in *Bacillus* spore coats

Software Authors
J.D. Manton[1], E.J. Rees[1]*
1 Department of Chemical Engineering and Biotechnology, University of Cambridge, CB2 3RA, UK.
* Correspondence ejr36@cam.ac.uk

This software is open source, and can be obtained from the following website (or alternatively by contacting Eric Rees with the correspondence address above). This source may be updated from time to time.
http://laser.ceb.cam.ac.uk/research/our-software

A version of the software from the time of publication is archived at data.cam.ac.uk/

LINK: https://www.repository.cam.ac.uk/handle/1810/251135

reference ID: [opendata #2026] at info@data.cam.ac.uk

Supporting Information 3: Model leading to equation 4 (spherical shell image)

Algebraic model for image of a spherical spore shell — EJR/HDR.

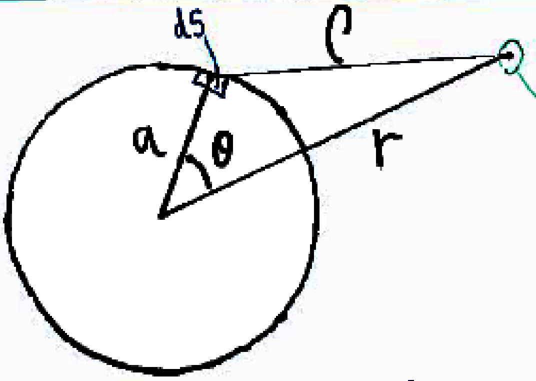

$f(r)$ = Image brightness at this point

ρ = Distance to area element dS

a = Spherical shell radius.

(1) $$\rho^2 = r^2 + a^2 - 2ar\cos\theta. \quad \text{(Cosine rule)}$$

(2) $$df = (I_o\, dS) \times \frac{e^{-\rho^2/2\sigma^2}}{(2\pi\sigma^2)}$$

- df: CONTRIBUTION TO IMAGE INTENSITY $f(r)$
- $(I_o\, dS)$: FLUORESCENCE EMITTED FROM AREA ELEMENT dS
- $\frac{e^{-\rho^2/2\sigma^2}}{(2\pi\sigma^2)}$: SPHERICALLY SYMMETRIC GAUSSIAN POINT SPREAD FUNCTION (NORMALISED).

(Integrate) $$f(r) = \left(\frac{I_o}{2\pi\sigma^2}\right)\int_0^{2\pi} d\phi \int_0^{\pi} a^2 \sin\theta\, d\theta\; e^{-(r^2+a^2-2ar\cos\theta)/2\sigma^2}$$

Substitute $x = \cos\theta$, $\theta = \pi \rightarrow x = -1$

$dx = -\sin\theta\, d\theta$, $\theta = 0 \rightarrow x = 1$

(Substitute) $$\left(\frac{1}{I_o}\right) f(r) = \left(\frac{a}{\sigma}\right)^2 \int_{-1}^{1} e^{-(r^2+a^2)/2\sigma^2}\, e^{+2arx/2\sigma^2}\, dx$$

(Separate) $$= \left(\frac{a}{\sigma}\right)^2 e^{-(r^2+a^2)/2\sigma^2} \int_{-1}^{1} e^{2arx/2\sigma^2}\, dx$$

(Integrate exponential) $$= \left(\frac{a\sigma^2}{r\sigma^2}\right) \cdot e^{-(r^2+a^2)/2\sigma^2} \cdot \left[e^{2ar/2\sigma^2} - e^{-2ar/2\sigma^2}\right]$$

(Difference of two squares →) $$f(r) = \left(\frac{aI_o}{r}\right)\left[e^{-(r-a)^2/2\sigma^2} - e^{-(r+a)^2/2\sigma^2}\right]$$

Supporting Information 4

Sample image data from this study is uploaded at data.cam.ac.uk/

LINK: https://www.repository.cam.ac.uk/handle/1810/251135
